# Ruptured Ovarian Endometrioma During Pregnancy: A Case Report and Review Highlighting Clinically Occult Risk Factors

**DOI:** 10.7759/cureus.101871

**Published:** 2026-01-19

**Authors:** Yumiko Miyazaki, Sakurako Takada, Rena Yamazaki, Shinya Hirabuki, Hiromasa Sasaki

**Affiliations:** 1 Obstetrics and Gynecology, Ishikawa Prefectural Central Hospital, Kanazawa, JPN

**Keywords:** acute abdomen, adhesions, endometrioma, pregnancy, rupture

## Abstract

The rupture of an ovarian endometrioma during pregnancy is a rare cause of acute abdominal pain, and its underlying risk factors are incompletely understood. We report a case of ovarian endometrioma rupture during pregnancy complicated by dense intra-abdominal adhesions and review previously reported cases to clarify potential risk factors. Twenty cases, including our case, were analyzed. Among cases with available data, 78.5% involved cysts measuring ≥6 cm, consistent with previous reports identifying large cyst size as a major risk factor. Adhesions were reported in seven cases, although most studies did not explicitly comment on their presence or absence. Regarding gestational age, 50% of cases occurred during the third trimester, and all cases presenting at ≥32 weeks of gestation were delivered by cesarean section. Beyond visible risk factors, such as cyst size and gestational age, clinically occult factors, including intra-abdominal adhesions, may also contribute to rupture risk. Careful early assessment and documentation of ovarian endometriomas may support clinical decision-making; however, the preventive role of pre-pregnancy surgical intervention warrants further investigation.

## Introduction

The rupture of an ovarian endometrioma during pregnancy is a rare but potentially serious cause of acute abdominal pain. Although ovarian endometriomas are common in women of reproductive age, rupture during pregnancy is uncommon, with only sporadic reports of such cases in the literature [1,2]. Symptoms are often nonspecific, and the differential diagnosis includes adnexal torsion, hemorrhagic cyst rupture, appendicitis, and other causes of acute abdominal pain during pregnancy [3]. Spontaneous hemoperitoneum in pregnancy (SHiP) is another rare cause of acute abdomen during pregnancy, reported in association with endometriosis-related vascular disruption [4], and should be considered in the differential diagnosis. Accordingly, the preoperative diagnosis of ovarian endometrioma rupture remains challenging in many cases.

Previous case reports have primarily focused on clinically identifiable risk factors such as cyst size and gestational age [1,3]. However, less attention has been paid to factors that are difficult to evaluate preoperatively, such as intra-abdominal adhesions [1,3,5].

We present a case of ovarian endometrioma rupture during pregnancy complicated by dense intra-abdominal adhesions and provide a review of previously reported cases. The objective of this study was to explore both clinically apparent and occult risk factors contributing to ovarian endometrioma rupture during pregnancy.

## Case presentation

Our patient was a 39-year-old woman, gravida 2, para 1, with no notable medical history. She had never been diagnosed with endometriosis. She conceived spontaneously and visited a local clinic at 16 weeks of gestation. However, she did not receive regular antenatal care and was referred to our institution for perinatal management at 30 weeks of gestation. No ovarian enlargement was apparent at the initial visit.

The patient developed sudden-onset, persistent upper abdominal pain at 33 weeks and 2 days of gestation. The pain did not resolve, and she was therefore transported to the emergency department. Upon admission, her vital signs were stable, with a body temperature of 37.2 °C, blood pressure of 114/73 mmHg, heart rate of 84 beats/min, and oxygen saturation of 98% on room air. Abdominal examination revealed tenderness in the right upper quadrant. Obstetric assessment revealed a reassuring fetal heart rate pattern with irregular uterine contractions. Vaginal examination showed a closed cervix without evidence of vaginal bleeding or rupture of membranes.

**Figure 1:**
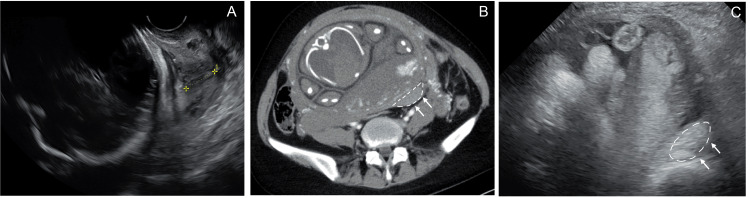
Ultrasonographic and computed tomographic findings of ruptured ovarian endometrioma during pregnancy. (A) Transvaginal ultrasonography revealed cervical shortening. (B) Transabdominal ultrasonography image showing a hematoma posterior to the uterus. The bleeding site is indicated by dotted lines and arrows. (C) Contrast-enhanced abdominal computed tomography image showing a high-density area posterior to the uterus, suggestive of a hematoma (indicated by dotted lines and arrows).

Laboratory investigations revealed a white blood cell count of 12,900/μL, a C-reactive protein level of 0.16 mg/dL, and a hemoglobin level of 10.9 g/dL (Table 1). Transvaginal ultrasonography demonstrated a shortened cervix of 18 mm (Figure 1A). Transabdominal ultrasonography revealed a hematoma posterior to the uterus (Figure 1B). Contrast-enhanced computed tomography revealed hemorrhagic ascites and a hematoma posterior to the left side of the uterus (Figure 1C). Based on these findings, the patient was diagnosed with spontaneous hemoperitoneum in pregnancy (SHiP).

**Table 1:** Laboratory findings at the time of diagnosis. WBC: white blood cell, Hb: haemoglobin, PLT: platelet count, T-Bil: total bilirubin, AST: aspartate aminotransferase, ALT: alanine aminotransferase, CK: creatine kinase, BUN: blood urea nitrogen, Cre: creatinine, CRP: C-reactive protein.

Parameter	Value	Reference range
WBC (/μL)	12,700	3300–8600
Hb (g/μL)	10.8	11.6–14.8
PLT (×10^3^/μL)	350	158–348
T-Bil (mg/dL)	0.4	0.4–1.5
AST (U/L)	16	13–30
ALT (U/L)	10	7–23
CK (U/L)	25	41–153
BUN (mg/dL)	5.0	8–20
Cre (mg/dL)	0.43	0.46–0.79
Amylase (U/L)	130	44–132
CRP (g/dL)	0.16	0–0.14

The patient was treated with acetaminophen and pentazocine; however, her abdominal pain worsened. Follow-up transabdominal ultrasonography revealed an increase in intra-abdominal fluid volume, prompting an emergency cesarean section with hemostatic surgery.

**Figure 2:**
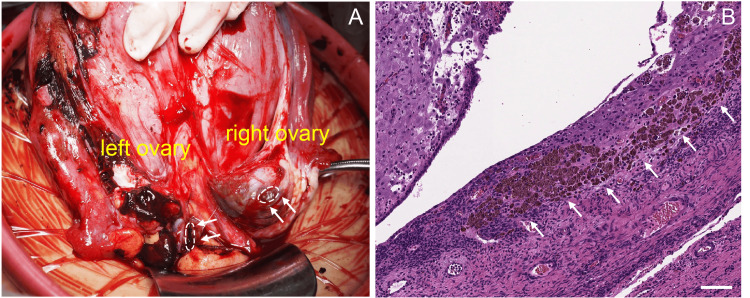
Intraoperative and histopathological findings of ruptured ovarian endometrioma. (A) Intraoperative findings showing bilateral ovarian endometriomas with cyst rupture on both sides (dotted lines and arrowheads indicate rupture sites). (B) The cyst wall was lined with endometrial tissue and showed hemosiderin deposition (indicated by arrows), and the endometrial stroma exhibited decidual-like changes. Scale Bar: 100 μm.

Hemorrhagic ascites were observed intraoperatively in the abdominal cavity. A female infant weighing 2016 g was delivered by cesarean section, with Appearance, Pulse, Grimace, Activity, and Respiration (APGAR) scores of 8 and 9 at 1 and 5 min, respectively [6]. The umbilical arterial pH was 7.21. Both ovaries contained endometriomas, and ruptured cysts with spilled contents were observed bilaterally (Figure 2A). The ovaries were densely and bilaterally adherent to the posterior uterine wall. Bilateral ovarian cystectomy was performed, followed by hemostasis and extensive peritoneal lavage. The estimated blood loss was 610 mL. Histopathological examination confirmed bilateral ovarian endometriomas (Figure 2B).

The patient’s abdominal pain resolved after surgery, and inflammatory marker levels gradually normalized. The postoperative course was uneventful, and she was discharged on day 6 after the operation. The neonate was admitted to the neonatal intensive care unit and discharged on day 29. This study was conducted in accordance with the principles of the Declaration of Helsinki. Written informed consent for publication of this report was obtained from the patient.

## Discussion

The rupture of an ovarian endometrioma during pregnancy is a rare but clinically serious cause of acute abdominal pain [3,7]. The risk factors for and mechanisms underlying ovarian endometrioma rupture are not fully understood owing to the rarity of the condition. Management strategies are mainly based on case reports and small case series. We present a case of ruptured ovarian endometrioma complicated by dense intra-abdominal adhesions, along with a review of 19 previously reported cases, summarized in Tables 2, 3, to explore both visible and clinically occult risk factors for rupture. Cases of SHiP without documented rupture of an ovarian endometrioma were excluded from this review because vascular disruption-related hemorrhage represents a distinct clinical entity that differs in pathophysiology and clinical course.

**Table 2:** Clinical characteristics of ovarian endometrioma rupture during pregnancy. Clinical characteristics, presenting symptoms, preoperative diagnosis, and gestational age at rupture in reported cases of ovarian endometrioma rupture during pregnancy, including the present case). AIH, artificial insemination by husband; ART, assisted reproductive technology; NR, not reported; S, spontaneous conception; SHiP, spontaneous hemoperitoneum during pregnancy.

Authors	Age (years)	Gravida	Para	Mode of conception	History of endometriosis	Gestational age (weeks)	Presenting symptoms	Preoperative diagnosis
Brill et al. [8]	38	3	2	NR	NR	NR (term)	Abdominal pain	Placental abruption
Steinberg [9]	35	1	0	S	No	38	Abdominal pain	Ruptured ovarian cyst
Anderson and Edmond [10]	39	2	1	NR	No	37	Abdominal pain	NR
Rossman et al. [11]	25	2	0	NR	NR	30	Abdominal pain with hyperpyrexia	Ruptured appendix
Johnson and Woodruff [12]	39	NR	NR	NR	NR	26	Lower abdominal pain	Degeneration of uterine fibroid
Vercellini et al. [13]	29	1	0	S	No	35	Abdominal pain	Bowel obstruction
Barbazan et al. [14]	29	1	0	NR	No	18	Lower abdominal pain with nausea and vomiting	NR
García-Velasco et al. [15]	25	NR	0	S	No	9	Lower abdominal pain	Hemorrhagic corpus luteum or endometrioma
Loh et al. [16]	25	NR	NR	S	NR	6	Lower abdominal pain	Ectopic pregnancy
Gregora and Higgs [17]	44	2	0	S	Yes	18	Lower abdominal pain	NR
Ueda et al. [18]	35	NR	NR	ART	NR	NR (second trimester)	NR	NR
Reif et al. [7]	25	NR	0	ART	Yes	27	Abdominal pain	NR
Yu et al. [19]	NR	NR	NR	NR	NR	35	Abdominal pain	NR
Takami et al. [1]	30	1	0	ART	Yes	10	Abdominal pain	Peritonitis
Takami et al. [1]	31	3	0	NR	NR	31	Abdominal pain	Ruptured endometrioma
Takami et al. [1]	32	1	0	NR	NR	32	Abdominal pain with vomiting	Acute appendicitis
Takami et al. [1]	34	2	1	NR	Yes	Postpartum	Abdominal pain	Ruptured endometrioma
Yamamoto et al. [2]	39	1	0	AIH	Yes	27	Abdominal pain	Ruptured endometrioma
Tanabe et al. [20]	43	3	1	NR	Yes	36	Abdominal pain	Placental abruption
Present case	39	2	1	S	No	33	Upper abdominal pain	SHiP

**Table 3:** Cyst characteristics as well as management and outcomes of ovarian endometrioma rupture during pregnancy. Cyst size, enlargement during pregnancy, surgical approach, presence of adhesions, and obstetric outcomes of reported cases of ovarian endometrioma, including the present case. For bilateral ovarian involvement, the ruptured side is specified if data were available. The bilateral cyst sizes are separately listed. NR, not reported; CS, cesarean section.

Authors	Side	Cyst size (cm)	Enlargement during pregnancy (cm)	Initial management	Surgical approach	Procedure	Adhesions	Gestational age at delivery (weeks)	Mode of delivery
Brill et al. [8]	Bilateral ovaries	NR	NR	Surgical	CS	Hysterectomy and bilateral salpingo-oophorectomy	NR	NR	Emergency CS
Steinberg [9]	Left ovary	6	NR	Surgical	CS	Cystectomy	Yes	38	Emergency CS
Anderson and Edmond [10]	Left ovary	NR	NR	Surgical	CS	Left oophorectomy	NR	37	Emergency CS
Rossman et al. [11]	Bilateral ovaries (side not specified)	NR	NR	Conservative	Laparotomy	Bilateral cystectomy	Yes	30	Vaginal
Johnson and Woodruff [12]	Right ovary	NR	NR	NR	CS	Cesarean section	NR	27	CS (type not specified)
Vercellini et al. [13]	Right ovary	8	NR	Surgical	CS	Cyst enucleation and adhesiolysis	Yes	35	Emergency CS
Barbazan et al. [14]	Right ovary	6	NR	Surgical	Laparotomy	Right oophorectomy	Yes	NR	NR
García-Velasco et al. [15]	Left ovary	8.3	6→8.3	Surgical	Laparotomy	Left salpingo-oophorectomy	Yes	NR	NR
Loh et al. [16]	Bilateral ovaries (left ruptured)	4 (left), 5 (right)	NR	Surgical	Laparoscopy	Bilateral cystectomy	Yes	39	NR
Gregora and Higgs [17]	Bilateral ovaries (left ruptured)	6 (left), 2.8 (right)	NR	Surgical	Laparotomy	Opening the cyst and stripping the cyst wall	NR	41	Vaginal
Ueda et al. [18]	Ovary (side not specified)	6	5→6	NR	Laparotomy	Peritoneal lavage and drainage	NR	NR	NR
Reif et al. [7]	Left ovary	NR	NR	Surgical	CS	Left salpingectomy and partial ovariectomy	Yes	27	Emergency CS
Yu et al. [19]	Right ovary	6	NR	Surgical	CS	Cystectomy	NR	35	Emergency CS
Takami et al. [1]	Left ovary	4	NR	Conservative	Laparotomy	Cystectomy, appendectomy, and drainage	NR	41	Vaginal
Takami et al. [1]	NR	5	NR	Surgical	Laparotomy	Abdominal drainage	NR	40	Vaginal
Takami et al. [1]	Right ovary	7	6→7	Surgical	CS	Cyst aspiration, cauterization, and appendectomy	NR	32	Emergency CS
Takami et al. [1]	Left ovary	10.5	9→10.5	Conservative	Laparotomy	Drainage and cystectomy	NR	41	Vaginal
Yamamoto et al. [2]	Right ovary	6.8	5→6.8	Conservative	CS	Cesarean section and cystectomy	NR	37	Elective CS
Tanabe et al. [20]	Left ovary	7	NR	Conservative	CS	Cystectomy	NR	36	Emergency CS
Present case	Bilateral ovaries	NR	NR	Surgical	CS	Cystectomy	Yes	33	Emergency CS

Currently, no consensus has been reached on the cyst size for which surgical intervention is recommended prior to pregnancy to prevent rupture during gestation. In our review, the mean maternal age was 33.4 years, and 68.7% of the patients were nulliparous (Table 2). Cysts measuring ≥ 6 cm accounted for 78.5% of the cases with available data in this review (Table 3), which is consistent with findings from reports identifying larger cysts as a visible risk factor for rupture during pregnancy [20]. Gestational age may also play a critical role in cyst rupture [3]. In our review, 50% of the cases occurred during the third trimester (Table 2), and all cases presenting at or beyond 32 weeks of gestation were managed with cesarean section. These findings suggest that progressive uterine enlargement in late pregnancy restricts the operative field, necessitating cesarean section to enable treatment of ovarian endometrioma rupture (Table 3). However, immediate delivery is not always mandatory; reports include a case at 31 weeks that reached term following surgical drainage alone [1] and another at 27 weeks that was successfully managed conservatively with analgesics [2]. These findings emphasize the need for individualized management strategies tailored to gestational age, clinical severity, and the overall condition of both mother and fetus.

In contrast to the amount of information available on tumor size and gestational age as risk factors, limited information exists on intra-abdominal adhesions in ovarian endometrioma. Adhesions were explicitly described in seven cases, including the present case (Table 2); however, most reports did not mention the presence or absence of adhesions. Adhesions in such cases may be underreported in the literature because they are often difficult to detect or underestimated on routine preoperative imaging. However, several studies have suggested an association between adhesions and ovarian endometrioma rupture [1,3,5]. In our case, dense adhesions severely restricted ovarian mobility, resulting in localized mechanical stress on the cyst wall (Figure 2A). This mechanical stress or traction may have increased wall tension and predisposed the endometrioma to rupture, even without marked cyst enlargement. These observations suggest that adhesions are a clinically occult risk factor for endometrioma rupture during pregnancy.

In this case, intra-abdominal fluid accumulation was observed preoperatively, raising suspicion of SHiP. SHiP may be associated with hypovolemic shock due to massive hemorrhage [4]. In contrast, rupture of an ovarian endometrioma does not necessarily result in severe bleeding. Therefore, the temporal progression of vital signs, perioperative findings, and the presence or absence of anemia may aid in differentiating between these two conditions. The proposed role of adhesions in endometrioma rupture is consistent with the pathophysiology of SHiP, in which deep infiltrating endometriosis-related fibrosis and adhesions are thought to induce traction-related vascular rupture during pregnancy [4,5]. Although decidualization has also been suggested to play a role in the development of SHiP [4], the relationship between decidualization and the enlargement or rupture of endometrioma remains largely unclear. While SHiP primarily involves vascular disruption rather than cyst wall rupture, both conditions may share a common mechanism through which anatomical pregnancy-related changes affect pre-existing endometriotic adhesions.

Our review highlights the difficulty in achieving an accurate preoperative diagnosis of ruptured ovarian endometrioma, with only 35.7% of cases correctly identified prior to surgery. Ruptured endometrioma should be considered in the differential diagnosis of acute abdominal pain during pregnancy given the nonspecific clinical presentation and limitations of prenatal imaging, particularly in patients with known or suspected endometriomas.

**Figure 3:**
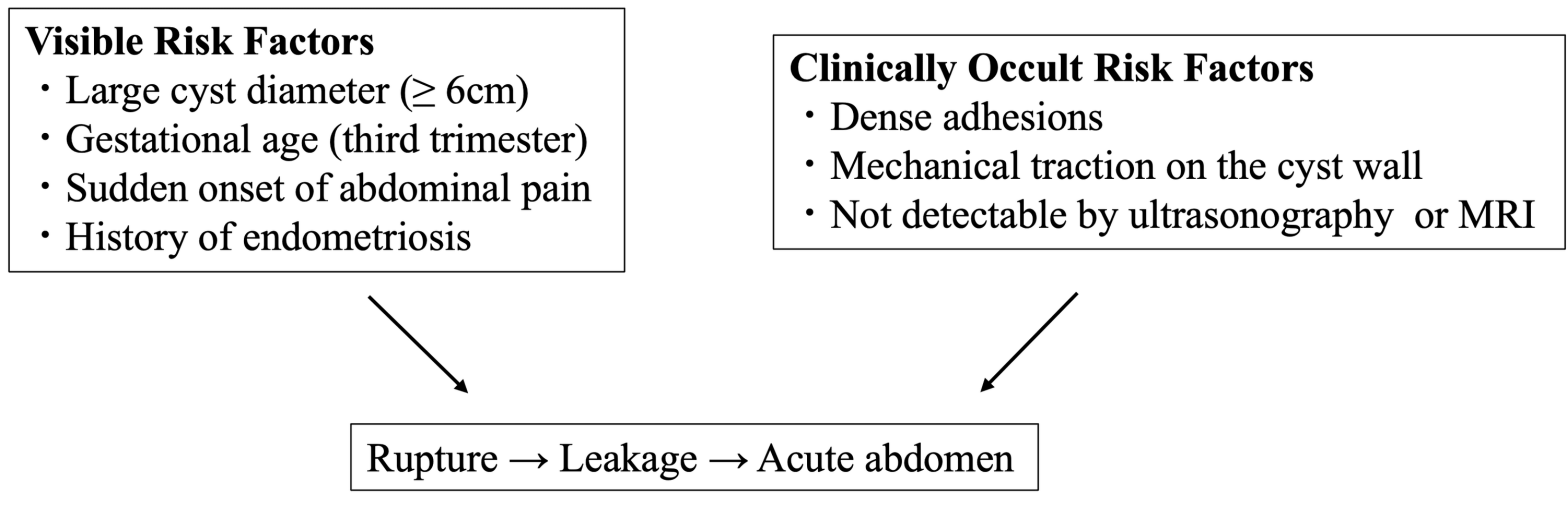
Conceptual framework of risk factors associated with ovarian endometrioma rupture during pregnancy. This schematic diagram summarizes the visible and clinically occult risk factors for ovarian endometrioma rupture during pregnancy based on the findings in this review. This figure illustrates a conceptual framework and does not imply definitive causal relationships.

Both visible and clinically occult risk factors should be considered when managing ovarian endometriomas during pregnancy (Figure 3). Early assessment for ovarian endometriomas may facilitate diagnosis. In select patients with large cysts and suspected adhesions, surgical intervention before pregnancy could reduce rupture-related complications.

This review is limited by the heterogeneity of the included case reports and the frequent absence of standardized or complete clinical data, which precludes robust quantitative analysis and necessitates cautious interpretation of the findings. Adhesions could not be objectively graded, and reporting bias cannot be excluded. However, highlighting adhesions as a clinically occult risk factor for ovarian endometrioma rupture provides a novel perspective for guiding the diagnosis and management of future cases and research.

## Conclusions

The rupture of an ovarian endometrioma during pregnancy is a rare but serious cause of acute abdominal pain. In addition to visible risk factors, such as cyst size and gestational age, clinically occult factors, such as intra-abdominal adhesions, may contribute to the risk of ovarian endometrioma rupture. Careful and early assessment and documentation of ovarian endometriomas may support clinical decision-making in these cases; however, the role of surgical intervention before pregnancy in preventing ovarian endometrioma rupture requires further investigation.
